# Mobilisation versus Bed Rest after Skin Grafting Pretibial Lacerations: A Meta-Analysis

**DOI:** 10.1155/2012/207452

**Published:** 2012-03-07

**Authors:** James Southwell-Keely, John Vandervord

**Affiliations:** Department of Plastic and Reconstructive Surgery, Royal North Shore Hospital, St Leonards, NSW 2065, Australia

## Abstract

Pretibial lacerations are problematic and best managed by surgical debridement, then skin grafting. Traditional postoperative care involves bed rest to optimise graft survival. This meta-analysis assesses early mobilisation versus bed rest for skin graft healing of these wounds. Medline, Embase, Cochrane, Cinahl, and Google Scholar databases were searched. Analyses were performed on appropriate clinical trials. Four trials met with the inclusion criteria. No difference was demonstrated in split skin graft healing between patients mobilised early compared to patients admitted to hospital for postoperative bed rest at either 7 (OR 0.86 CI 0.29–2.56) or 14 days (OR 0.74 CI 0.31–1.79). There was a statistically significant delay in healing in patients treated with systemic corticosteroids (OR 8.20 CI 0.99–15.41). There was no difference in postoperative haematoma, bleeding, graft infection, or donor site healing between the comparison groups. In the available literature, there is no difference between early mobilisation and bed rest for the healing of skin grafts to pretibial wounds. Corticosteroids exert a negative effect on skin graft healing unlike early mobilisation, which does not cause increased haematoma, bleeding, infection, or delayed donor site healing. Modality of anaesthesia does not affect skin graft healing.

## 1. Introduction

Pretibial lacerations are a common injury in the elderly often leaving nonviable traumatic skin flaps [1–3]. Intrinsic factors negatively impacting on the healing of pretibial lacerations include anatomical constraints, age-related changes, and vascular insufficiency [4, 5]. Proximal muscle bellies, that facilitate skin graft healing, give way to tendons distally, that provide a hostile environment for skin graft healing [6–8]. Anteriorly there is a paucity of subcutaneous tissue padding between the skin and the tibia, while the skin is fairly inelastic and with increasing age becomes thinner thus less resistant to trauma [9, 10]. Extrinsic factors affecting wound healing in pretibial lacerations may include diabetes mellitus, systemic corticosteroids, and malnutrition. The prevalence of systemic corticosteroid use in this population of patients is up to 40% [11].

Treatment options for pretibial lacerations include primary closure, defatting then resecuring the traumatic skin flap or debridement, and skin grafting. The former two options produce less predictable results [12–14]. Debridement and skin grafting involve the creation of a separate wound, but this donor site and the skin graft usually heal uneventfully.

Postoperatively dressings support the skin graft until healing is complete [4]. Traditional logic has held that skin grafts to the leg required five to seven days of bed rest with leg elevation to encourage healing without the burden of increased hydrostatic pressure in the leg of the erect patient [15]. Bed rest causes patient deconditioning and is a risk factor for venous thromboembolic disease [16, 17]. 

Bodenham and Watson first questioned the need for prolonged postoperative bed rest in 1971 [18]. In this case series, twenty-five patients underwent split skin grafting to the leg and were allowed to mobilise around the ward within 24–48 hours of the operation [18]. Eighty-four per cent of patients were healed by three weeks. Subsequent publications have reported differing results. A meta-analysis was performed to determine whether early mobilisation is as effective as bed rest for wound healing in patients split skin grafted for pretibial lacerations.

## 2. Methods

The meta-analysis was performed according to guidelines set out in the QUORUM statement [19].

### 2.1. Searching

A search of Medline, Embase, Cochrane, Cinahl, and Google Scholar was performed. Searches were performed using multiple combinations of Medical Subject Headings (MESH). Bibliographies of retrieved studies were crossed referenced. No non-English language trials were identified. No other published or unpublished data was identified upon consultation with experts in the field.

### 2.2. Selection

The published title and abstract of identified studies were assessed. Full text copies of the manuscripts were obtained for studies addressing the clinical question. The inclusion criteria were clearly identified patient population (split skin grafting to leg lacerations), intervention group (early mobilisation), comparison group (bed rest), and primary outcome (skin graft healing). Secondary outcomes assessed were corticosteroids induced delay in healing, reduced mobility, haematoma, bleeding, graft infection, time to donor site healing and healing at 7 and 21 days versus modality of anaesthesia.

### 2.3. Validity Assessment

Both randomised controlled trials and a combination of randomised controlled trials and prospective cohort studies were included in the analyses. Analyses including prospective cohort studies were performed to increase power, while sensitivity analyses confirmed that the results were not being corrupted with the inclusion of these patients. Nonclinical trials were excluded from the analyses. Methodological quality of the studies was assessed using the CONSORT Statement [20–22].

### 2.4. Data Abstraction

Studies were assessed for adequacy of randomisation, allocation concealment, blinding, similarity of treatment groups, similarity of care provided to the respective treatment groups other than the intervention of interest, intention to treat analysis, and the impact of losses to followup.

### 2.5. Study Characteristics

This meta-analysis assessed trials, both randomised and prospective cohort, in which patients split skin grafted for pretibial lacerations comparing early mobilisation with post-operative bed rest [23]. The primary outcomes were skin graft healing at 7 and 14 days.

### 2.6. Quantitative Data Synthesis

Odds ratios (OR) were calculated with 95% confidence intervals. Skin graft healing was reported both in terms of the percentage healing at 7 days and as a dichotomous outcome. Results reported as percentage healing were converted to dichotomous results using a one-to-four scoring system published by Wallenberg, where one signified primary healing of the whole graft, two signified the graft was healed, but with some minor defects, three signified 50% graft loss, and four signified essentially no graft take [24]. Grafts scoring one or two were classified as healed while those scoring three or four were classified as not healed. The same criteria were applied to skin graft healing at 14 days. Outcome statistics and forest plot diagrams were created using Revman 4.2 software [25]. Contact with the authors of the primary studies was attempted when missing data was identified. When no reply from the authors of the primary studies was received, sensitivity analyses were performed with the substitution of data in best and worst case scenarios. Heterogeneity of studies was assessed with the *χ* ^2^ and *I^2^* statistics [26]. Experts in the field were consulted in an attempt to identify unpublished data, the exclusion of which may have contributed to publication bias.

## 3. Results

### 3.1. Trial Flow

**Figure 1:**
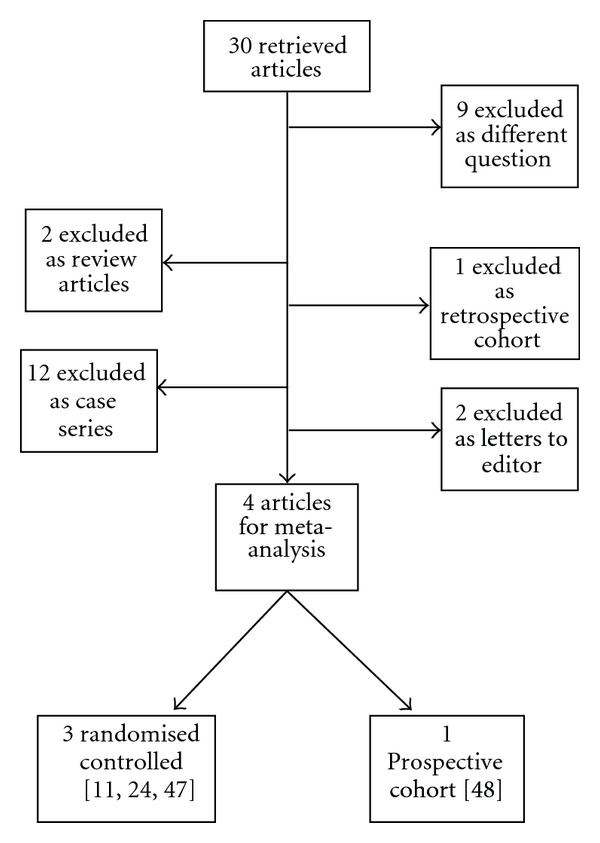
Meta-analysis flow diagram.

An extensive literature review retrieved 30 articles (see Figure 1).

There were four articles included in the meta-analyses entailing three randomised controlled trials and one prospective cohort study.

### 3.2. Study Characteristics

Appropriate studies were small in size with variable methodological quality. Analyses were performed both without and with data from the cohort study [23]. The remaining 26 studies were excluded from analysis (Figure 1) [27–46].

Budny et al. recruited 82 patients in 2 years, then excluded 21 leaving 61 patients for analysis [47]. It is not clear whether these patients were excluded before or after randomisation casting doubt upon the integrity of the randomisation process. Pseudo-randomisation was employed with the use of birth dates to determine treatment group allocation. Percentage skin graft healing was reported at 7 and 21 days. Wood and Lees report the results of a postal survey of 26 plastic surgery units in the United Kingdom concerning timing to recommence mobilisation after split skin grafting of pretibial wounds [11]. During 21 months, 75 patients were randomised into treatment and control groups. Percentage skin graft healing was reported at 7, 10, and 14 days. Wallenberg enrolled 50 consecutive patients requiring skin grafting, however only 9 of the 50 patients had sustained trauma to the lower limb, 4 in the early mobilizing group and 5 in the bed rest group [24]. Randomisation took place postoperatively by drawing slips of paper from a box. Graft healing was assessed at day 14 and scored subjectively. Gaze reported results of a prospective cohort study of 30 patients [48]. Graft healing was assessed at 7 days.

### 3.3. Quantitative Data Synthesis (Table 1)

#### 3.3.1. Primary Outcomes

**Skin Graft Healing**

The odds ratio for percentage skin graft healing at 7 days was −2.16 (95% CI −9.05–4.72) with low heterogeneity (*χ* ^2^ = 0.34, df = 1, *P* = 0.56 and *I^2^* = 0%) [11, 47]. The result was similar when expressed as a dichotomous outcome (OR = 0.86, 95% CI 0.29–2.56), with low heterogeneity (*χ* ^2^ = 0.02, df = 1, *P* = 0.89 and *I^2^* = 0%). Adding data from the prospective cohort study by Gaze et al. did not alter this result [11, 47, 48].

**Figure 2:**
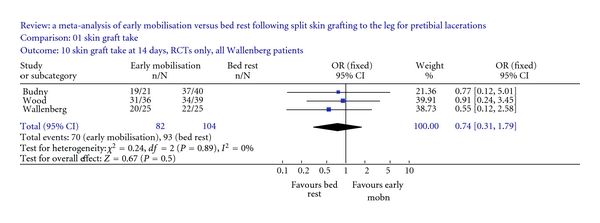
Meta-analysis of early mobilisation versus bed rest following split skin grafting to the leg for pretibial lacerations: skin graft take at 14 days—randomised controlled trials (RCTs) and all Wallenberg patients [11, 24, 47]. Odds ratio 0.74 (95% CI 0.31–1.79), *χ* ^2^ = 0.24, df = 2 (*P* = 0.89), *I^2^* = 0%.

The odds ratio for skin graft healing at 14 days was 0.86 (95% CI 0.29–2.56) with low heterogeneity (*χ* ^2^ = 0.02, df = 1, *P* = 0.89 and *I^2^* = 0%) [11, 24, 47]. Including all the Wallenberg patients in the analysis gave an odds ratio of 0.74 (95% CI 0.31–1.79) with low heterogeneity (*χ* ^2^ = 0.24, df = 2, *P* = 0.89 and *I^2^* = 0%) (Figure 2). Adding data from the prospective cohort study by Gaze to both analyses did not alter either result [48].

#### 3.3.2. Secondary Outcomes

**Healing Time and Systemic Corticosteroids**

Systemic corticosteroids delayed healing (OR 8.20, 95% CI 0.99–15.41) [11]. Mean time to healing in patients taking steroids was 31.50 days (SD 17.20) versus 23.30 days (SD 11.10) in patients not taking steroids.

**Postoperative Mobility**

A single study reported postoperative mobility results from 47 of the 61 patients enrolled in the trial [47]. None of the patients in the early mobilisation group experienced a reduction in mobility (0/16), however ten patients (10/31) in the bed rest group reported reduced mobility after hospitalization (OR = 0.06, 95% CI 0.00–1.14). Given the missing data, sensitivity analyses were performed as best (OR = 0.94, 95% CI 0.27–3.22) and worst (OR = 0.03, 95% CI 0.00–0.45) case scenarios.

**Other Secondary Outcomes**

The remaining secondary outcomes are reported in Table 1.

## 4. Discussion

In 1994, Wood and Lees reported the practice patterns of plastic surgery units in the United Kingdom for post-operative mobilisation protocols after split skin grafts to traumatic leg wounds [11]. A postal survey of 26 plastic surgery units had an 81% response rate. Less than 25% of patients were mobilised within 24 hours of skin grafting, while 57% of patients were confined to bed rest for five or more days. Early mobilisation after skin grafting the leg was first reported in the literature in 1971 by Bodenham et al., yet the practice remains to be widely employed [18, 27].

Pretibial lacerations present with varying degrees of severity, from linear lacerations amenable to primary closure, to extensive degloving injuries that may require pedicled or microvascular flap reconstruction. While both extremes of presentation provide a differing array of reconstructive challenges, they are beyond the scope of this meta-analysis.

Conclusions reached in this analysis may be scrutinised due to the small number, sizes, and the methodological quality of included trials. Randomisation techniques varied greatly and there was no mention made of allocation concealment. Blinding was only stated to have taken place in some of the trials. Reporting of the similarity between treatment and control groups at the commencement of the respective studies is variable. An intention to treat analysis appears to have taken place in each of the studies, yet this is not definitively stated. One of the trials may have reported postrandomisation exclusions and losses to follow up [47]. These shortcomings conspire to introduce bias to the results of the following analyses. There is also a risk of publication bias, which is inherent to all meta-analyses. Trials with negative findings are less frequently published, hence analyses tend to be performed upon primary trials with more dramatic results. This form of bias is less likely to be a factor in this meta-analysis since none of the primary articles showed a statistically significant difference between the two treatment groups.

At 7 days, healing of split skin grafts to pretibial lacerations was as effective with early mobilisation as with post-operative bed rest. Skin graft healing was reported as a percentage of the wound healed at 7 days (OR = −2.16, 95% CI −9.05–4.72) and as a dichotomous outcome with randomised controlled trial patients only and with the addition of participants from the prospective cohort study (OR = 0.86, 95% CI 0.29–2.56). There was no demonstrable difference in outcome between the two groups in either analysis.

At 14 days after surgery, no difference in the healing of split skin grafts to pretibial lacerations could be demonstrated between patients permitted to mobilise early compared to those who were confined to bed rest. Analyses including traumatic wounds from the trial by Wallenberg gave an odds ratio of 0.86 (95% CI 0.29–2.56), while including all of the Wallenberg patients gave an odds ratio of 0.74 (95% CI 0.31–1.79) [24]. The addition of patients from the prospective cohort study left this latter result unchanged [48]. As for 7 days there was no demonstrable difference in outcome between the two groups at 14 days.

By including patients from a prospective cohort study in the analyses, there is a risk of introducing bias into the results. Accordingly sensitivity analyses were performed both without and with this data. The results from these analyses were not changed. The situation would have been quite different had the inclusion of cohort study patients changed the results of the analyses. Had this been the case, one would be inclined to exclude these patients from the analyses.

Corticosteroids caused a statistically significant delay in healing (OR = 8.20, 95% CI 0.99–15.41). The mean time to wound healing in patients taking steroids was 31.50 days (SD 17.20) versus 23.30 days (SD 11.10) in patients not taking steroids. Patients dependent on systemic corticosteroids are a high risk group for problematic wound healing. Accordingly all available means to optimise healing should be employed to facilitate graft take in this subpopulation of individuals.

Reduction in mobility was reported to be statistically significant in the bed rest group compared with the early mobilisation group (OR = 0.06, 95% CI 0.00–1.14) [47]. The 14 losses to follow up in this trial account for 23% of the study population making interpretation of this result difficult. A worst case scenario analysis did not reach statistical significance (OR = 0.94, 95% CI 0.27–3.22), while a best case scenario analysis was statistically significant (OR = 0.03, 95% CI 0.00–0.45). At best, the stated differences between the treatment groups are an empiric observation, but little more can be gleamed from this conclusion.

There were no statistically significant differences between treatment groups in any of the remaining secondary analyses (Table 1).

## 5. Conclusions

In the available literature, there is no difference in the healing of split skin grafts to pretibial lacerations in patients managed with early mobilisation compared to patients managed with postoperative bed rest. Systemic corticosteroids delay the healing of pretibial wounds treated with split skin grafts. There is insufficient data in the published surgical literature to assess whether or not permitting early mobilisation spared the patient from reduced post-operative mobility. Early mobilisation was not associated with an increased incidence of haematoma, bleeding complications, infection, or delayed donor site healing when compared with bed rest. Deep vein thrombosis, pulmonary embolism, and chest infection were not noted to have occurred more frequently in either treatment group. Administration of general, regional, or local anaesthesia had no impact on the rate of skin graft healing among patients grafted for leg lacerations.

## Figures and Tables

**Table 1:** Primary and secondary outcomes.

	Study details	Studies	Patients	Odds ratio (95% CI)
Primary outcomes				
Graft healing at 7 days	RCTs	2	136	0.86 (0.29–2.56)
Graft healing at 7 days	RCTs + CS	3	166	0.86 (0.29–2.56)
Graft healing at 14 days	RCTs trauma patients [24]	3	145	0.86 (0.29–2.56)
Graft healing at 14 days	RCTs all patients [24]	3	186	0.74 (0.31–1.79)
Graft healing at 14 days	RCTs + CS trauma patients [24]	4	176	0.86 (0.29–2.56)
Graft healing at 14 days	RCTs + CS all patients [24]	4	216	0.74 (0.31–1.79)

Secondary outcomes				
Reduction in mobility	RCT	1	47	0.06 (0.00–1.14)
Reduction in mobility	RCT, worst case scenario [47]	1	61	0.94 (0.27–3.22)
Reduction in mobility	RCT, best case scenario [47]	1	61	0.03 (0.00–0.45)
Haematoma	RCT	1	61	0.95 (0.08–11.13)
Heparin coagulopathy	RCT	1	61	0.61 (0.02–15.69)
Graft infection	RCT	1	61	0.95 (0.08–11.13)
Delayed healing versus corticosteroids	RCT	1	75	8.20 (0.99–15.41)
Healing time versus anaesthesia	RCT	1	75	−4.60 (−10.88–1.68)
Donor site healing	RCT	1	75	−0.50 (−2.88–1.88)

Abbreviations: RCT, randomised controlled trials; CS, cohort study.
